# The identification of a novel interaction site for the human immunodeficiency virus capsid on nucleoporin 153

**DOI:** 10.1099/jgv.0.002104

**Published:** 2025-05-14

**Authors:** Shunji Li, Peik Lund-Andersen, Szu-Huan Wang, F. Marty Ytreberg, Mandar T. Naik, Jagdish Suresh Patel, Paul Andrew Rowley

**Affiliations:** 1 Department of Biological Sciences, University of Idaho, Moscow, ID 83844, USA; 2 Institute for Modeling Collaboration and Innovation, University of Idaho, Moscow, ID 83844, USA; 3 Department of Molecular Biology, Cell Biology, and Biochemistry, Brown University, Providence, RI 02912, USA; 4 Department of Physics, University of Idaho, Moscow, ID 83844, USA; 5 Department of Chemical and Biological Engineering, University of Idaho, Moscow, ID 83844, USA

**Keywords:** capsid, HIV-1, nuclear import, nucleoporin 153, NUP153

## Abstract

Human immunodeficiency virus type-1 (HIV-1) can infect non-dividing cells by passing through the selective permeability barrier of the nuclear pore complex. The viral capsid is essential for successfully delivering the HIV-1 genome into the nucleus. Nucleoporin 153 (NUP153) interacts with the HIV-1 capsid via a C-terminal capsid-binding motif (hereafter named CbM.1) to licence HIV-1 nuclear ingress. Deletion or mutation of CbM.1 in NUP153 causes a reduction in capsid interaction but does not prevent HIV-1 nuclear ingress or completely block capsid interaction. This paper combines molecular modelling with biochemical and HIV infection assays to identify capsid-binding motif 2 (CbM.2) in the C-terminus of NUP153 that is similar in sequence to CbM.1. CbM.2 has an FG dipeptide motif predicted to interact with a hydrophobic pocket in capsid protein (CA) hexamers similar to CbM.1. CA hexamers can interact with CbM.2, and the deletion of both CbM.1 and CbM.2 results in a lower capsid interaction than a single CbM.1 deletion. The loss of CbM.1 is complemented by CbM.2, an interaction dependent on the FG motif. In the context of the nuclear pore complex, a loss-of-function mutation in CbM.1 reduces HIV nuclear ingress as measured by transduction and 2-LTR circles, whereas the mutation of CbM.2 causes a large increase in 2-LTR circles. Our results highlighted a previously unidentified FG dipeptide-containing motif (CbM.2) in NUP153 that binds the HIV-1 capsid at the common hydrophobic pocket on CA hexamers.

## Introduction

Passage through the nuclear pore complex (NPC) is an essential step in the human immunodeficiency virus (HIV) replication cycle, enabling HIV integration into the human genome. The monomeric HIV capsid protein (CA) oligomerizes to form the characteristic fullerene cone of HIV (referred to hereafter as ‘capsid’), which is constructed of hexamers and pentamers [1]. The capsid is essential for shepherding the HIV genome to the nucleus, allowing reverse transcription and shielding from innate immune sensors [2,3]. Although many HIV proteins have the potential to be transported through the NPC to the nucleoplasm [4,6], experiments have shown that the capsid plays a dominant role in enabling the infection of non-dividing cells [7]. Several models seek to explain the molecular mechanism of HIV nuclear ingress and the structure of the capsid as it is transported through the NPC [8,10]. The degree to which the capsid is remodelled and the role of host proteins in this process remain to be fully characterized.

HIV nuclear ingress requires many host proteins, including the NPC [11,15]. Many proteins of the NPC (nucleoporins) are important for the HIV replication cycle, including those with repeat sequences of the amino acids phenylalanine (F) and glycine (G) (NUP358/RanBP2, NUP153, NUP214, NUP62, NUP98 and POM121) [16,23]. These ‘FG’ nucleoporins are localized to different positions in the NPC and have different patterns of FG repeats. Specifically, the depletion of nucleoporin 153 (NUP153) reproducibly affects the HIV replication cycle by reducing HIV nuclear ingress [17,19,23] and alters the site of HIV integration to regions of heterochromatin [19,26]. NUP153 is anchored to the nuclear basket and has a mostly disordered structure that can protrude from the nucleoplasm into the cytoplasm [27,30]. In addition, NUP153 is also a mobile nucleoporin with varying residence times at the nuclear basket [30]. NUP153 can interact with many different host proteins, including other components of the NPC, can transport factors that regulate the movement of cargo between the nucleus and cytoplasm [31,32] and plays a role in the DNA damage response [33]. Like many nucleoporins that fill the central channel and project from the surface of the NPC, the C-terminal domain of NUP153 (NUP153c) contains phenylalanine-glycine (FG) repeat motifs. FG repeat motifs are thought to provide interaction sites for the transient binding of cellular transport proteins and block the free diffusion of large macromolecules through the nuclear pore by forming a dynamic permeability barrier [34,36].

Given the diversity of FG patterns in nucleoporins, how they aid in HIV-1 infection is poorly understood, particularly regarding the specificity of FG repeats in these nucleoporins and their interaction with HIV-1. A recent study investigated the binding affinity between HIV-1 CA and FG-domains of NUP98, NUP214, NUP62, POM121 and NUP358/RanBP2 and found they can be co-precipitated with *in vitro* assembled HIV-1 CA oligomers [25]. These interactions suggest that nucleoporins can directly interact with HIV-1 CA, but how this relates to the process of nuclear entry remains unresolved. It has been proposed that nuclear ingress by the capsid could mimic cellular nucleocytoplasmic transport by karyopherins, which involves multiple weak but specific interactions [34,36]. However, in NUP153, one FG motif has been shown to bind with high affinity to a hydrophobic pocket formed between two CA monomers in the context of a hexamer and bind with lower affinity to CA monomers [17,37]. For clarity, we have named this region on NUP153c ‘capsid-binding motif 1’ (CbM.1) (_1407_TNNSPSGVFTFGANSST_1423_). In CbM.1, the central amino acids from P1411–G1418 are most important for interacting with the capsid, with the F1417 phenyl group intercalating into the hydrophobic pocket of the capsid [17,25, 37, 38]. There are also other residues flanking the central FG motif that are also important for stable association with the capsid [37,38]. In addition to NUP153, the cellular proteins CPSF6 and SEC24C interact with CA hexamers, using phenylalanine residues to bind the same hydrophobic pocket as NUP153. However, the residues surrounding FG motifs in CPSF6 and SEC24C interact with different CA residues compared to the CbM.1 motif of NUP153 [37,39]. The importance of this pocket as an essential docking site for cellular proteins during the HIV replication cycle has led to an interest in the targeting of the capsid as a therapeutic intervention. The occlusion of the hydrophobic pocket has been repeatedly shown to be effective at preventing HIV nuclear ingress. Small molecules that target this interface have also been used to block the HIV replication cycle [40,44]. The development of these molecules has been successfully translated into an effective therapeutic [45,46]. For NUP153, only a complete deletion of the C-terminal domain causes the total loss of CA binding [17,25]. Single mutations and small deletions do not abolish capsid interaction [17,37]. This suggests that there are additional interaction sites present within NUP153, and recently, a non-FG motif-rich triple-arginine motif has been identified to interact with the capsid [22]. However, given the relative simplicity and small size of the CbM.1 sequence in NUP153c and the large number and diversity of FG motifs in nucleoporins, it is plausible that there are other motifs in cellular proteins with the potential to interact with the HIV capsid that remain to be discovered.

In this study, we have successfully identified a new motif with sequence similarity to CbM.1 in NUP153 that can interact with the hydrophobic pocket formed between two CA monomers. As confirmed by molecular modelling studies, this new motif in NUP153 has a similar binding pose to CbM.1 and a similar, albeit slightly lower, affinity for CA tubes. NUP153 mutants that replace CbM.1 with capsid-binding motif 2 (CbM.2) can still interact with CA tubes and with capsid in a TRIM-NUP153 restriction assay. In the context of the nuclear pore complexes, depletion of NUP153 and complementation with GFP-NUP153 with mutations disrupting CbM.1 and CbM.2 showed that both are important for the completion of the HIV replication cycle. Specifically, the loss of CbM.1 resulted in the expected reduction in HIV transduction and 2-LTR circles, indicating a block to nuclear ingress. Surprisingly, the loss of CbM.2 resulted in a slight increase in transduction but a large and disproportionate increase in 2-LTR circles, which indicated a possible block to HIV integration that remains to be explored further.

## Methods

### Cells and viruses

HEK293T cells, HeLa cells and CRFK cells were maintained at 37°C with 5% CO_2_ in Dulbecco’s Modified Eagle Medium (DMEM) (Sigma-Aldrich #D6429) supplied with 10% FBS (Sigma-Aldrich), 2 mM l-glutamine (VWR #L0131-0100) and 1% penicillin/streptomycin solution (Corning, #30–002). CRFK cells transduced with MLV carrying TRIM-NUP153C mutations were cultured in the same conditions as other cell lines, except for the addition of a final concentration of 3 µg/ml puromycin (Thermo Fisher # A1113803) to DMEM. Single-cycle HIV-1 virus with a GFP reporter gene was generated as previously described [38]. Single-cycle HIV-1 carrying firefly luciferase (HIV-Luc) was generated in HEK293T cells under the same conditions except that plasmids used were pCMV-VSV-G, pHAGE-CMV-Luc2-IRES-ZsGreen (NR-52516), pHDM-CMV-Gag-Pol (NR-52517), pHDM-CMV-Tat1b (NR-52518) and pRC-CMV-Rev1b (NR52519). EIAV-GFP and EIAV-Luc were created by transfecting a 10-mm dish of HEK293T cells with 4 µg of pEV53D (Addgene # 44168), 4 µg of pCMV-VSV-G and 4 µg of the transfer plasmids [pEIAV-SIN6.1CBLucWm1A (Addgene # 44163) for EIAV-Luc and pEIAV-SIN6.1 CGFPW (Addgene #44171) for EIAV-GFP]. TRIM-NUP153C-containing MLV was produced by transfecting HEK293T cells in a 6-well plate, with 1 µg of pCS2-mGP, 0.2 µg of pC-VSVG and 2 µg of the pLPCX vector carrying TRIM-NUP153C and desired mutants. Viruses were titrated on HEK293T (HIV-GFP), followed by flow cytometry (Beckman Coulter) or on HeLa (HIV-Luc and EIAV-Luc), followed by luciferase assays, respectively. Viruses were snap-frozen with liquid nitrogen and then stored at −80°C.

### Plasmids and primers

The plasmid pLPCX-TRIM-NUP153C (human)-HA encoding the TRIM domain from TRIM5α of Rhesus macaque (residues 1 to 304) fused to the HA-tagged human NUP153 C-terminal domain (896 to 1475) was obtained from the Engelman laboratory [17]. TRIM-NUP153C-HA was amplified by PCR and sub-cloned to the Gateway™ entry vector pCR8 to create plasmid pUI034. Gateway™ cloning introduced the gene into the custom destination vector pCDNA3-GW, following the manufacturer’s instructions (Thermo Fisher). All plasmids used in this study can be found in Table S1, available in the online Supplementary Material. Primers used in site-directed mutagenesis were designed at NEBaseChanger® (https://nebasechanger.neb.com/). All primers used in this study can be found in Table S2.

### Molecular modelling

Molecular dynamics simulation and *in silico* mutagenesis of NUP153 by FoldX were performed as previously described [38]. To model the other three NUP153 motifs, modeller software was used to engineer residues into the input structure of the WT HIV-1 CA hexamer (PDB ID:4U0D) and build the missing residues in all the chains to complete the experimental structure [47]. During Molecular dynamics (MD) simulation, the input structure, including all three NUP153 motifs, was subjected to atomistic MD simulation using the protocol reported in our previous study [48]. Briefly, the AMBER99SB*-ILDNP force field and the GROMACS 2018.3 software package [61] for the motifs, with the amber-99sb-star-ildnp force field, were used for generating topology files and performing simulations [49,51]. The final production simulation was run for 100 ns, and snapshots were saved every 1 ns, resulting in 100 snapshots for the protein complex. The MD trajectory was visualized using the VMD software package and analysed using the grmsf module available in the GROMACS package to calculate the root mean square fluctuation (RMSF) of all the atoms in each residue in the NUP153 motif during the simulation [52]. FoldX software was used to estimate the relative binding affinities (ΔΔG_bind_) for all possible mutations at each site in the NUP153 motif as previously described [38].

### Surface plasmon resonance

The binding affinity of peptides to CA hexamers was measured by surface plasmon resonance (SPR) using the Biacore X100 (Cytiva). The CM5 sensor chip (Cytiva) was pre-conditioned with 100 mM HCl at a 30 µl min^−1^ flow rate. Purified monomeric CA hexamers protein was immobilized to a surface density of approximately 7,400 response units by using the Amine Coupling Kit (1-ethyl-3-(3-dimethylaminopropyl) carbodiimide hydrochloride/*N*-hydroxysuccinimide, Cytiva). Experiments were conducted at 25°C, in PBS pH 7.4, 0.005% [v/v] Tween-20, 5% (v/v) DMSO. NUP153 motif peptides and a negative scrambled control (SNFPTNSAVSNSGTGF) were synthesized by GenScript (Piscataway, NJ) and dissolved in 100% DMSO. Peptide binding was tested by injecting series concentrations (6.25 µM to 800 µM) at a flow rate of 30 µl min^−1^ and measured by multi-cycle determinations. Raw data were analysed by the Biacore X100 evaluation software (version 2.0.1) and fitted to the steady-state affinity.

### RNA interference and NUP153 complementation

HeLa cells were seeded at 1×10^5^ ml^−1^ in a 24-well dish. After 24 h, cells were transfected with 10 nM siRNA duplexes/well using 3 µl Lipofectamine RNAiMAX (Invitrogen # 13778075). siNup153: GCAUCGCCGAAGAUAGAUUtt [53]; scrambled: AUUAGCUAUCGAGAGCGCAtt (Sigma-Aldrich). At 48 h post-transfection, cells were seeded onto a 96-well chimney plate (Greiner # 655098) and transfected with 250 ng of siRNA-resistant pEGFP(C3)-NUP153 (Addgene #64268) and mutants using Lipofectamine 3000 (Invitrogen # L3000001) [27]. Cells were transduced with HIV-luc or EIAV-luc 24 h post-transfection. Expression of complementing NUP153 was examined by flow cytometry using the mean fluorescent intensity as a readout.

### Luciferase assays

Luciferase assays (Promega # E2610) were performed after 48 h post-transduction, along with a 96-well format Bradford assay (Alfa Aesar # J61522-AP) (both on SpectraMax iD3, Molecular Devices). Specifically, the supernatant was removed, and cells were washed with 1×PBS pH 7.4. After the PBS was aspirated, 100 µl of luciferase reagent (1 : 1 diluted in 1× PBS pH 7.4) was added to each well. The plate was allowed to incubate at room temperature and occasionally patted until the cells were all lysed. Each cell had 5 µl of cell lysate withdrawn to be subject to the Bradford assay performed alongside the luciferase assay. In a 96-well plate (Falcon # 08-772-54), each examining well contained 95 µl of Bradford reagent and 5 µl of cell lysate. A standard curve was generated along with each assay. For data analysis, the total protein amount in each well was calculated based on the standard curve. The raw relative light unit (RLU) of each well was then converted to RLU µg^−1^ protein.

### CA tube co-sedimentation assay

These assays were performed as described previously [38] and were adapted from Selyutina *et al*. [54], involving seeding ~200,000 HEK293T cells per well in a 12-well plate. After 24 h of incubation, cells were transfected with 500 ng of pCDNA3-TRIM-NUP153C using 1.5 μl of TransIT-293 transfection reagent (Mirus Bio). Following another 24-h incubation, cells were harvested by scraping into 100 μl of CA binding buffer (10 mM Tris, pH 7.4, 1.5 mM MgCl_2_, 10 mM KCl and 1× Halt protease and phosphatase inhibitor cocktail; Thermo Fisher, PI78440). The lysates were mixed at 4°C for 15 min and then clarified by centrifugation at 21,000 ***g*** for 15 min at 4°C. Protein concentrations were standardized to 1.5 mg ml^−1^ using a Bradford assay. Next, 20 μl of CA tubes (~1.4 μM) were combined with 80 µl of whole-cell lysate and incubated at room temperature for 1 h. The mixtures were centrifuged at 21,000 ***g*** for 8 min at 4 °C, and 15 µl of the supernatant was collected for analysis using Western dot blotting.

### Cell flow cytometry and TRIM-NUP153C-mediated restriction

This assay was modified from Matreyek *et al*. [17]. HEK293T cells (200,000 per well) were seeded and transfected with TRIM-NUP153C following the protocol described for the CA co-sedimentation assay. Twenty-four hours post-transfection, recombinant HIV-GFP viral stocks, supplemented with 8 μg ml^−1^ polybrene, were titrated to achieve ~30% GFP-positive cells. The culture medium was replaced with fresh medium 24 h after transduction. Forty-eight hours post-transduction, cells were treated with 0.05% trypsin (VWR, 16777-202) and centrifuged at 2,000 ***g*** for 3 min at room temperature. Cell pellets were suspended and fixed in 300 μl of Dulbecco’s PBS (DPBS) (VWR, 45,000-434) containing 1% paraformaldehyde (Electron Microscopy Sciences, 15710) and incubated at 4°C for 1 h. Following fixation, cells were centrifuged at 2,000 ***g*** for 3 min, and the cell pellets were washed twice with 500 μl DPBS. The cells were then suspended in 100 μl flow cytometry buffer (DPBS with 4% FBS) and transferred to a 96-well U-bottom assay plate (Celltreat, 229590). Fluorescent cells were quantified using the CytoFLEX S flow cytometer (Beckman Coulter).

### Western blotting

Samples were resolved by SDS-PAGE and transferred to nitrocellulose membranes using the Trans-Blot Turbo Transfer System (1.0 A, 25 V, 15 min). Alternatively, samples were applied directly to nitrocellulose membranes via a Bio-Dot microfiltration apparatus (Bio-Rad). Membranes were blocked with 3% nonfat milk in TBS containing 0.1% Tween 20 (TBST) for 1 h. For HA tag detection, membranes were incubated with rat anti-HA-HRP antibody (3F10, Sigma-Aldrich, 12013819001; 1:2,000 dilution) for 1 h. To detect CA and actin, membranes were treated with rat anti-p24 antibody (ARP-6457, NIH HIV Reagent Program; 1 : 5,000 dilution) or rat anti-actin antibody (VWR, 10221-880; 1 : 500 dilution) for 1 h. Following primary antibody incubation, membranes were washed three times with 5 ml of TBST for 5 min each while gently rocking. Anti-p24 blots were then transferred to a fresh tray and incubated with goat anti-rat antibody (Thermo Fisher, 62-652-0; 1 : 4,000 dilution) for 40 min. Blots were visualized, and signal intensities were quantified using an Amersham Imager 600. Exposure times were manually adjusted to 10 s for anti-HA blots, 4 s for anti-p24 blots and 10 s for anti-actin blots.

### Statistical analysis

All analyses for assays were performed using Prism 9 software.

## Results

### Loss of CbM.1 does not prevent NUP153 interaction with the capsid

**Fig. 1.**
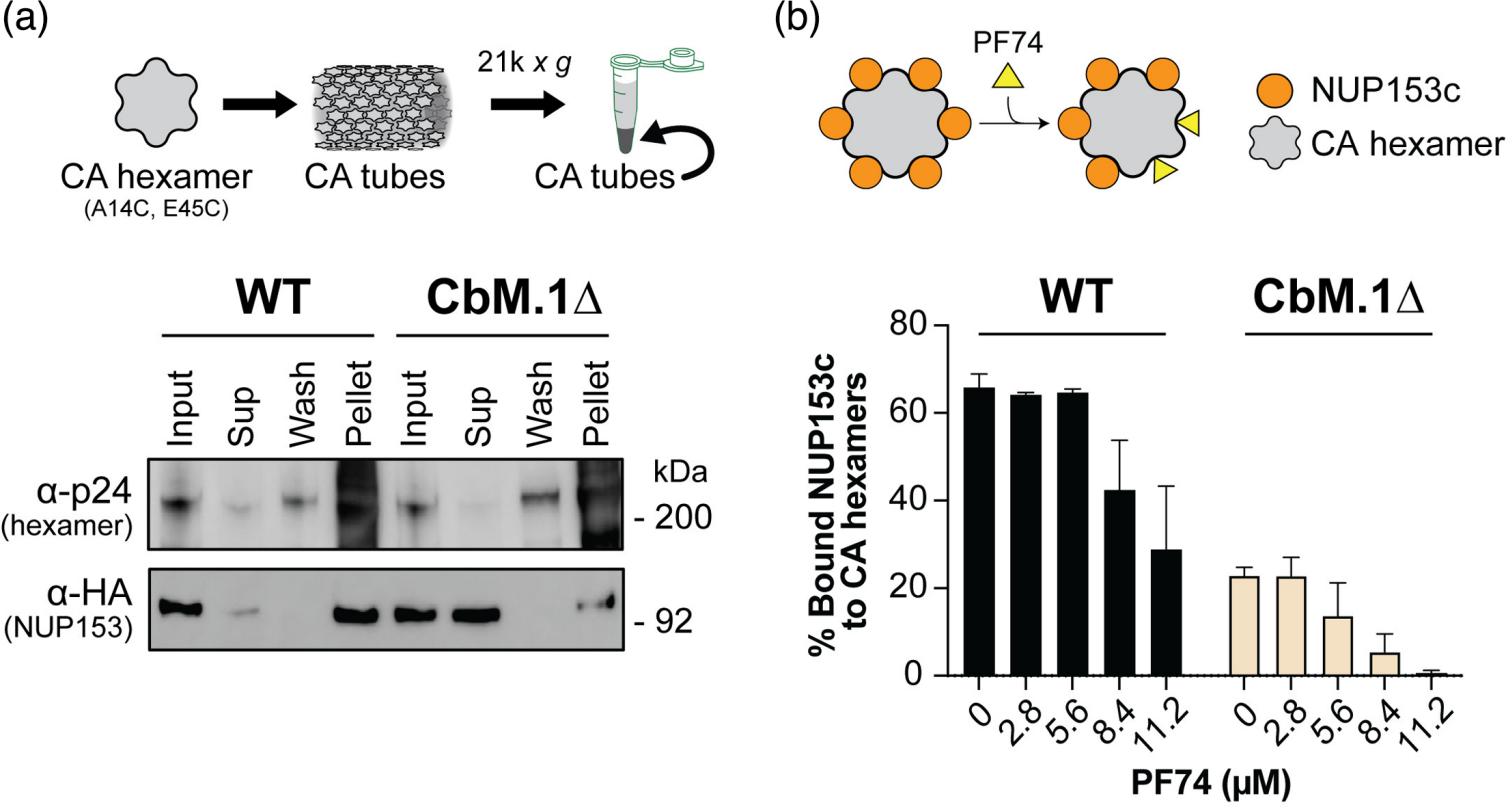
NUP153 binds CA hexamers without CbM.1, an interaction blocked by PF74. (**a**) Top. A schematic detailing the co-sedimentation of NUP153c with high molecular weight CA hexamer tubes. Bottom. The co-sedimentation of WT NUP153c and CbM.1∆ with HIV CA hexamers. Western blotting detected HIV p24 and HA-tagged NUP153c in the whole cell extract (Input), supernatant (Sup), rinsate of the pellet (Wash) and Pellet fractions. Expression of NUP153c was induced by HEK293T transfection with 1 µg plasmid per well (200,000 cells), and the protein content of lysates for Western blotting was normalized by Bradford assay to ~4 µg/well. The blot is a representation of three independent biological replicate blots. (**b**) Top. A schematic of the interaction of PF74 with the hydrophobic pockets of the CA hexamer showing the displacement of NUP153c. Bottom. Quantification of the interaction between NUP153c or CbM.1∆ with CA hexamer tubes in the presence of increasing amounts of PF74. Error bars are standard error (*n*=4, biological replicates).

We first asked whether CbM.1 was the only capsid-binding site on NUP153. To test this, we assembled the disulfide-stabilized CA hexamers (A14C/E45C) into large molecular weight tubes and used them to co-sediment WT NUP153c or the CbM.1∆ mutant *in vitro*. Briefly, lysates from cells expressing WT NUP153c or the mutant were clarified by centrifugation and incubated with CA hexamer tubes. After centrifugation, the proportion of NUP153c associated with the sedimented CA tubes was determined by quantitative Western dot blotting [38]. Deletion of CbM.1 (CbM.1∆) caused a reduction of CA hexamer tube interaction (Fig. 1a), but ~20% of CbM.1∆ remained associated with CA hexamer tubes after co-sedimentation (Fig. 1b, 0 µM PF74). This association depended on the CA hexamer tube assembly (Fig. S1). The binding of both WT and CbM.1∆ to the CA hexamer tubes was disrupted by the addition of PF-3450074 (PF74), which binds to the same hydrophobic pocket as CbM.1 and stabilizes hexameric capsid tubes at concentrations ~10 µM (Fig. 1b) [55]. These data suggested that CbM.1∆ binds to the CA hexamer tubes at the same hydrophobic pocket as CbM.1, indicating the presence of an alternative capsid-binding motif in NUP153.

### FG-containing motifs in NUP153 homologous to CbM.1 are predicted to bind the capsid

**Fig. 2.**
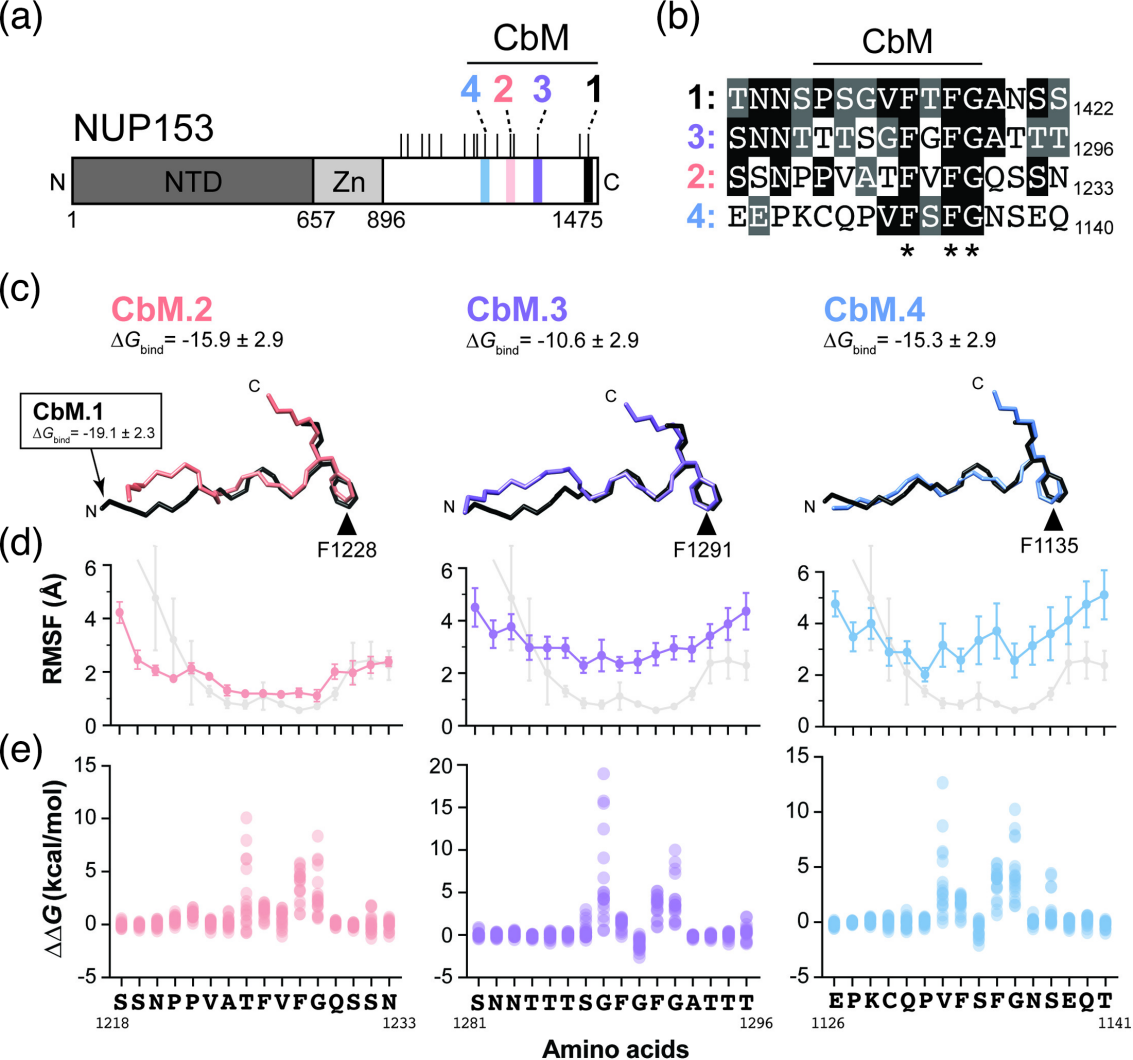
The molecular modelling of four FxFG-containing motifs in NUP153 bound to the HIV capsid. (**a**) A protein domain diagram of NUP153 (aa 1–1475) showing FxFG motifs with homology to CbM.1. Tick marks represent FxFG repeats. Zn, zinc finger domain. NTD, N-terminal domain. (**b**) The alignment of three FxFG motifs with sequence similarity to CbM.1. Black or grey shading represents >50% identity or similarity to CbM.1, respectively. (**c**) A peptide backbone representation of CbM.2 (left, pink), CbM.3 (middle, purple) and CbM.4 (right, blue) bound to CA and superimposed on the peptide backbone of CbM.1 (black) (PDB:4U0C). The phenylalanine R-group of the ‘FG’ motif is represented in all models as a point of reference. (**d**) Average RMSF of each amino acid residue of the four CbM.1 homologues bound to a CA hexamer during a 100 ns MD simulation. For comparison, CbM.1 RMSF is shown in grey (*n*=6). Error bars represent the standard deviation. (**e**) ΔΔG_bind_ calculated by MD combined with FoldX for all possible substitutions at each amino acid position in the three CbM.1 homologues.

To look for possible capsid-binding motifs on NUP153, we searched for CbM.1-homologous sequences using blast and identified three motifs in NUP153c that were named CbM.2 (1,222–1,229 aa), capsid-binding motif 3 (CbM.3) (1,285–1,292 aa) and capsid-binding motif 4 (CbM.4) (1,129–1,136 aa) (Fig. 2a). All motifs are surrounded by low-complexity regions with a paucity of charged residues and an overabundance of phenylalanine, glycine, serine and threonine residues (Fig. S2). These motifs also contained a central FxFG sequence that in CbM.1 is vital for the interaction with the hydrophobic pocket of the HIV capsid (Fig. 2b). CbM.1 and CbM.3 flank a predicted prion-like domain with CbM.2 and CbM.4 positioned in a non-prion forming region toward the N-terminal domain (Fig. S2).

MD simulations were carried out to determine whether CbM.2, CbM.3 and CbM.4 can interact with CA hexamers and to observe the stability of their interactions (Fig. 2c). CbM.2 and CbM.4 had the lowest values of calculated free energy of binding. CbM.2 was more stably bound to the CA binding pocket, with an average RMSF of 1.89 Å, compared to CbM.3 (3.22 Å) and CbM.4 (3.61 Å) (Fig. 2d). *In silico* mutagenesis using FoldX combined with MD simulations found that the FG motif was critical for the interaction of CbM.2, CbM.3 and CbM.4 with capsid and the residue preceding the FxFG motif (Fig. 2e). Together, computational predictions indicated that the CbM.1 homologues may bind to the hydrophobic pocket of CA with similar binding poses.

### CbM.2 influences NUP153 binding to CA tubes

**Fig. 3.**
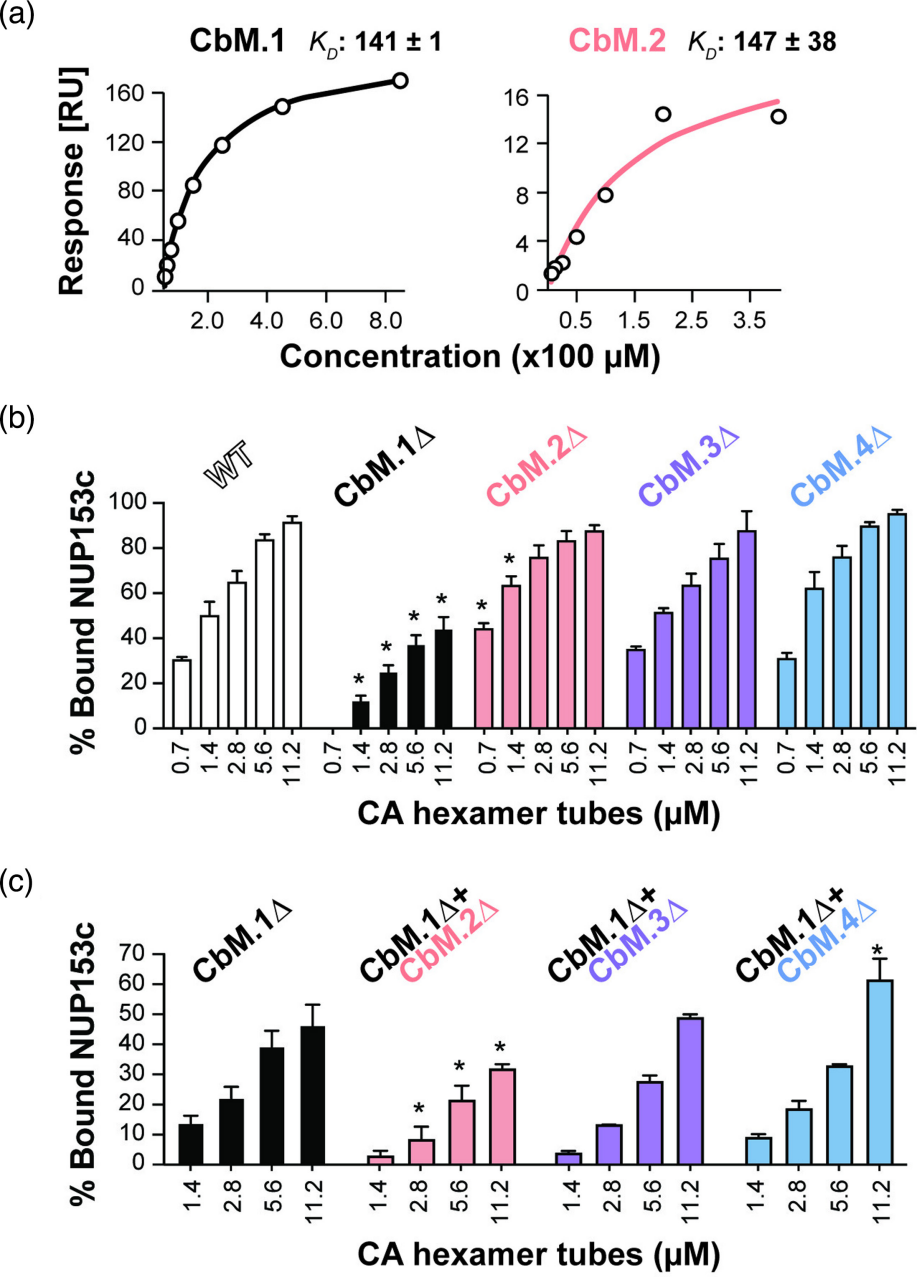
CbM.2 interacts with HIV CA hexamers. (**a**) SPR analyses of CbM.1 and CbM.2 peptide binding to CA hexamers. *K_D_* calculated for 1 : 1 binding (*n*=3, biological replicates). (**b–c**) Co-sedimentation of CA tube interaction with (**b**) single and (**c**) double deletion mutants. Expression of NUP153c and the mutants was induced by HEK293T transfection with 0.5 µg plasmid per well (100,000 cells), and the protein content of lysates for Western blotting was normalized by Bradford assay to ~2 µg/well. The assays included three biological replicates. Asterisks: significant difference from (**b**) WT and (**c**) CbM.1∆ (*P*<0.05, Tukey’s test). Error bars are standard error.

To validate these CA hexamer-NUP153 modelling predictions, 16-mer peptides of each CbM were used to test binding to recombinant CA hexamers using surface plasmon resonance. Consistent with previously published findings, CbM.1 had a *K_D_* of 141 µM±1 µM, and CbM.2 had a *K_D_* of 147±38 µM (Figs 3a and S3) [56]. In contrast, the *K_D_* of CbM.4 (2870±840 µM) and a scrambled CbM.2 (450±284 µM) had much larger *K_D_* values, and CbM.3 binding could not be calculated (Figs 3a and S3). We then focused on understanding the binding between the CbMs and the capsid in the context of the NUP153c protein. Overall, the individual deletion of these motifs did not diminish NUP153c binding to assembled CA tubes, as assayed by co-sedimentation. Surprisingly, more CbM.2∆ co-sedimented compared to WT NUP153c but only at the two lowest concentrations of CA tubes assayed (Fig. 3b). To avoid confounding effects associated with the presence of CbM.1 and its high affinity for CA tubes, double deletion mutants were created with CbM.1 and either CbM.2, CbM.3 or CbM.4. Co-sedimentation assays demonstrated that CbM.1∆/CbM.2∆ association with CA tubes was significantly reduced compared to CbM.1∆ alone and the two other double mutants tested (CbM.1∆/CbM.3∆ and CbM.1∆/CbM.4∆) (Fig. 3c). Together, these data suggest that together with CbM.1, CbM.2 interacts with CA tubes.

### CbM.2 can complement the loss of CbM.1

**Fig. 4.**
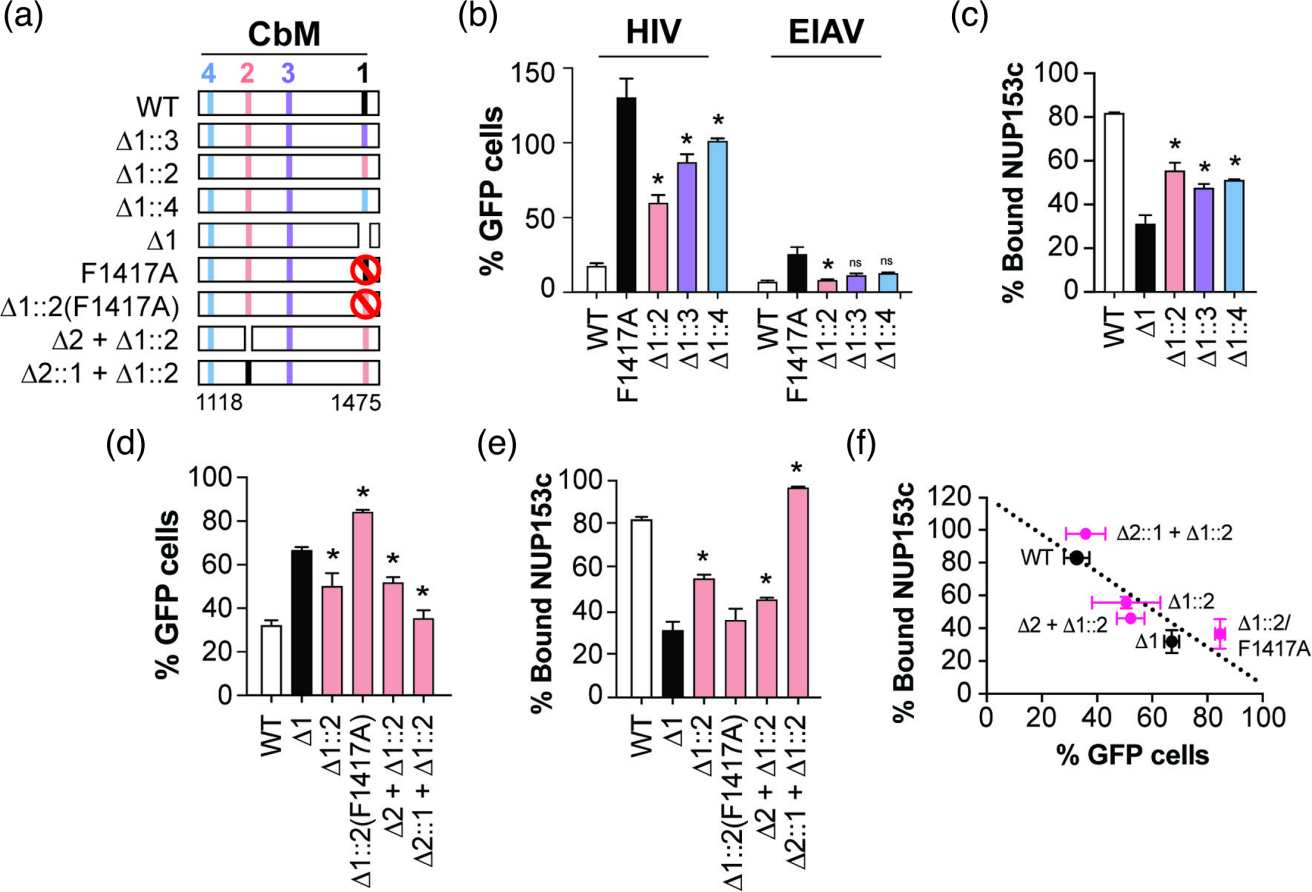
CbM.2 can complement CbM.1 in the C-terminus of NUP153. (**a**) A representation of aa 1118–1475 in NUP153c showing the position of CbM.1, CbM.2, CbM.3 and CbM.4. NUP153c mutants with different configurations of these motifs were designated as ∆1 (CbM.1∆), ∆1::2 (CbM.1∆ complemented with CbM.2∆), ∆1::3 (CbM.1∆ complemented with CbM.3∆), ∆1::4 (CbM.1∆ complemented with CbM.4∆), ∆1::2(F1417A) (CbM.1∆ complemented with CbM.2∆ with the mutation F1417A), ∆2 + ∆1::2 (CbM.2∆ with CbM.1∆ complemented with CbM.2) and ∆2::1 + ∆1::2 (CbM.2∆ complemented with CbM.1 and CbM.1∆ complemented with CbM.2). Tick marks represent FxFG repeats. (**b and d**) A cell-based restriction assay with stable (**b**) or transient (**d**) expression of TRIM-NUP153c mutants transduced by HIV-GFP and EIAV-GFP. Virus transduction was measured relative to an empty vector control. (**c and e**) CA hexamer tube co-sedimentation assays with NUP153c mutants. All assays were repeated independently at least three times. Asterisks indicate a significant difference from NUP153c F1417A (**b**) or CbM.1∆ (**c-e**) using Tukey’s test (*P*<0.05). (**f**) Correlation of data from panels (d) and (e) [*R*^2^=0.72 (*P*<0.0001)].

As the loss of CbM.1 caused a dramatic loss of capsid interaction, we next asked whether CbM.2, CbM.3 or CbM.4 could functionally replace CbM.1. Therefore, we created TRIM-NUP153c constructs where CbM.1 was replaced by different CbMs (Fig. 4a). When being transduced by HIV-GFP, TRIM-NUP153c blocks HIV-GFP transduction by directly binding to the capsid via NUP153c, resulting in reduced GFP signals. As expected, WT NUP153c reduced the number of HIV-transduced GFP-positive cells to 18.5% relative to empty vector control, indicating direct NUP153c interaction with capsid (Fig. 4b). This contrasts the loss-of-binding mutation F1417A in CbM.1 that caused an approximately sevenfold increase in GFP-positive cells, which is consistent with a loss of capsid interaction (Fig. 4b). HIV restriction was best after the replacement of CbM.1 with CbM.2 (∆1::2, Fig. 4b), relative to F1417A in CbM.1. Replacement of CbM1 with CbM.3 or CbM.4 reduced HIV restriction (∆1::3 and ∆1::4, Fig. 4b), but was not judged to be due to poor expression of TRIM-NUP153c (Fig. S4). However, all TRIM-NUP153c proteins were active restriction factors and fully capable of restricting EIAV-GFP, which is dependent on a motif between aa 924–927 (Fig. 4b) [17]. Finally, the loss of capsid interaction after the deletion of CbM.1 could also be partially restored by replacement with CbM.2 (∆1::2), CbM.3 (∆1::3) or CbM.4 (∆1::4) as measured by co-sedimentation with CA hexamer tubes (Fig. 4c).

As CbM.2 had consistently shown to be important for capsid interaction, the loss-of-binding mutation in the phenylalanine of the FG motif in CbM.2 was used to substitute CbM.1∆ (∆1::2(F1417A)). This mutation resulted in the loss of NUP153c interaction demonstrated by TRIM restriction similar to CbM.1∆ (Fig. 4d) and the loss of capsid hexamer tube co-sedimentation (Fig. 4e). These data again show that CbM.2 interacts with CA and validate prior molecular models and mutagenesis experiments (Fig. 2). The substitution of CbM.1∆ by CbM.2 was independent of CbM.2 at its WT position (1,218–1,233 aa), as the loss of CbM.2 (CbM.2∆) with CbM.2 complementing CbM.1∆ (∆2 + ∆1 : 2) only slightly reduced co-sedimentation with CA tubes and did not reduce TRIM-NUP153c restriction (Fig. 4d, e). To test whether the positioning of CbM.1 and CbM.2 in NUP153c was important for capsid binding, the two motifs were swapped (∆2::1 + ∆1::2). In this configuration, capsid binding was similar to WT NUP153c as assayed by TRIM restriction assay and co-sedimentation (Fig. 4d, e). This would indicate that the presence of CbM.1 at the canonical CbM.2 position (1,218–1,233 aa) could fully restore the interaction between NUP153 and capsid. However, this is in contrast to (∆2 + ∆1::2) and ∆1::2, which only partially restored capsid interaction, indicating a dominant role of CbM.1 in capsid interaction compared to CbM.2. Differences in the expression of the various NUP153c mutants were also measured by Western blotting and could not explain the differences in CbM.1 complementation (Fig. S5). Furthermore, we observed no significant differences between these mutants when transducing cell lines with EIAV-GFP, except for ∆1::2(F1417A), which appeared to have caused a loss of restriction (Fig. S6). These data together showed that CbM.2, not CbM.3 or 4, can functionally complement CbM.1 in interaction with the capsid. Comparing the directly binding to the capsid by NUP153c and the percentage GFP from the TRIM assay are in good agreement with an *R*^2^ of 0.72 (*P*<0.0001) (Fig. 4f).

### CbM.1 deletion reduces HIV-1 nuclear ingress, whereas CbM.2 deletion causes an increase in 2-LTR circles

**Fig. 5.**
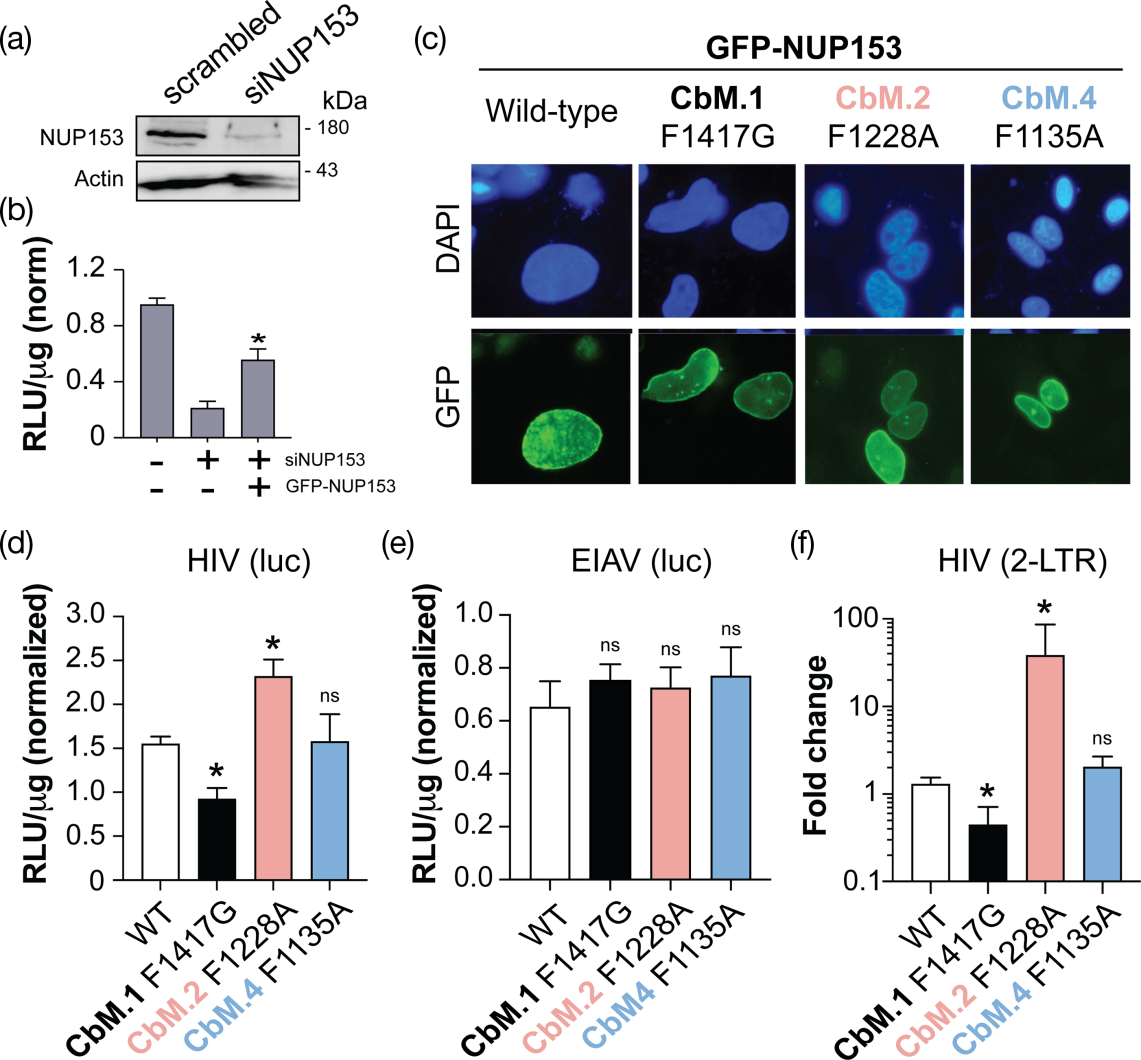
The loss of CbM.1 or CbM.2 specifically alters HIV nuclear ingress. (**a**) The depletion of NUP153 by siRNA was measured by Western blotting. (**b**) HIV transduction in WT and NUP153 knockdown cell lines with and without complementation by WT and siRNA-resistant GFP-NUP153. (**c**) The localization of GFP-NUP153 in four complemented cell lines compared to DAPI staining of nuclei as viewed under a light microscope. The effect of NUP153 complementation by WT and mutant GFP-NUP153 on (**d**) HIV-luciferase transduction, (**e**) EIAV-luciferase transduction and (**f**) 2-LTR circles. To measure HIV and EIAV transduction ((**d**) and (**e**)) using a luciferase reporter, the total protein amount was first calculated based on the standard curve. The raw relative light unit (RLU) of each well was then converted to RLU/µg protein and normalized to an empty vector control. All assays included at least three biological replicates. Asterisks indicate a significant difference from WT GFP-NUP153 complemented cell line using Tukey’s test (*P*<0.05); ns indicates no significant difference from WT.

To test the importance of CbM.1 and other homologous motifs during virus nuclear entry, endogenous NUP153 was depleted in HeLa cells using siRNA (Fig. 5a). The depletion of NUP153 resulted in an ~80% reduction of luciferase signal after being transduced by HIV-luciferase compared to the scrambled siRNA control cells (Fig. 5b). The knockdown of endogenous NUP153 was successfully rescued by the transfection of a plasmid expressing siRNA-resistant GFP-NUP153, resulting in nuclear and perinuclear GFP localization (Figs 5c and S7). Transfection with plasmids encoding GFP-NUP153 with predicted loss-of-binding mutations in CbM.1 (F1417G), CbM.2 (F1228A) and CbM.4 (F1138A) showed similar numbers of GFP-positive cells and fluorescence compared to WT GFP NUP53 (Fig. S8). These mutants also localized to the nucleus when used to rescue the loss of endogenous NUP153 (Fig. 5c). As expected, WT GFP-NUP153 expression resulted in an approximately twofold increase in HIV transduction relative to the NUP153-depleted cells (Fig. 5d). In contrast, CbM.1 (F1417G) complemented the loss of endogenous NUP153 poorly, with 20% less luciferase signal than WT GFP-NUP153. Surprisingly, CbM.2 (F1228A) increased the luciferase signal by 50% (Fig. 5d). The disruption of CbM.4 (F1135A) did not alter HIV transduction compared to WT GFP-NUP153 (Fig. 5d), which is consistent with the previous data showing a lack of capsid interaction (Figs3  4). The complementation of NUP153 depletion with either WT or mutant GFP-NUP153 did not alter EIAV-luciferase transduction under the same assay conditions (Fig. 5e), suggesting that the loss of CbM.1 and CbM.2 specifically affects HIV. As an alternative measure of HIV nuclear ingress, 2-LTR circles were monitored by quantitative PCR. Consistent with HIV transduction data (Fig. 5d), complementation by CbM.1 (F1417G) resulted in a 60% reduction in 2-LTR circles, indicating a block to nuclear ingress. Conversely, complementation by mutant CbM.4 (F1228A) was indistinguishable from WT NUP153 (Fig. 5f). Surprisingly, mutant CbM.2 (F1228A) was found to increase 2-LTR circles by 30-fold, a phenotype often associated with efficient nuclear entry and defects in HIV integration.

## Discussion

This study demonstrated that HIV-1 capsid interacts with more than one FxFG motif in NUP153c. The previously documented FxFG-containing motif (CbM.1; 1,411–1,418 aa) interacts with HIV-1 CA hexamers at a hydrophobic pocket formed by NTD helices 3 and 4 and packs against hydrophobic side chains [37]. Although CbM.1 is important for the interaction with CA hexamers, loss of this motif does not abolish NUP153 binding, as deletion of aa 1410–1417 or the F1415A mutation can still interact with capsid [17]. The residual interaction between NUP153 and capsid appears specific to the same hydrophobic pocket bound by CbM.1 and is different from an interaction of a NUP153 triple-arginine motif with capsid [22]. The minimal capsid binding motif was previously predicted by empirical and molecular modelling studies as a relatively short sequence of eight amino acids [37,38]. Surprisingly, despite the prevalence of FG repeats in human nucleoporins, homologues of CbM.1 were only identified in the C-terminus of NUP153 and were not found in other proteins associated with the NPC.

Molecular modelling of FxFG motifs that are homologous to CbM.1 (CbM.2, CbM.3 and CbM.4) was predicted to interact with CA hexamers in a similar binding pose as CbM.1. The phenylalanine residue of the ‘FG’ motif served as an anchor to enable binding to the hydrophobic pocket in CA hexamers. As expected, the *in silico* mutation of the FG dipeptide was disruptive to the capsid interaction of CbM.2, CbM.3 and CbM.4, consistent with empirical experiments and modelling studies with CbM.1. A recent study demonstrated that in human primary monocyte-derived macrophages, passing HIV-1 capsid through the NPC resulted in the cracking of the NPC rings [57]. This remodelling of the NPC ring may potentiate an alternative set of FG nucleoporins (and/or FG motifs), compared to the intact NPCs, to access the capsid. Indeed, the potential of all CbMs to interact with the HIV capsid is consistent with studies showing that many FG nucleoporins can also interact with the HIV capsid [35,36]. This suggests that movement through the NPC relies on multivalent interactions with FGs, consistent with our data showing that NUP153 can still weakly bind CA tubes even without CbM.1 and CbM.2. However, each CbM likely interacts with the HIV capsid differently, which is demonstrated by the different behaviours and binding affinities of the CbM deletion and substitution mutants in our assays. Different binding poses and affinities are known features of FG-containing HIV cofactors [37,39].

The residues in NUP153 surrounding the FG motif in CbMs were also crucial in determining the interaction with CA residues at and around the hydrophobic interface, as shown by the mutational analysis of CbM.1 [37,38]. These data are consistent with *in silico* mutagenesis of CbM.2, CbM.3 and CbM.4, which show the importance of flanking residues for capsid interaction. Moreover, a prion-like domain flanking CbM.1 is thought to play a role in capsid interaction by contributing to binding avidity [58]. CbM.2 is positioned away from this domain and would not benefit from such additional interactions for capsid binding, and CbM.1 can interact with the capsid when moved to the canonical CbM.2 position. These data suggest that the lack of the prion-like sequence does not negatively affect CA-CbM.1 interaction. The interaction studies demonstrate that CbM.2 is a novel capsid-interacting motif. However, it is important to recognize that CA tube binding assays and TRIM restriction assays do not address the biological relevance of CbM.2 or CbM.1 in the context of the NPC. *In vitro* assays would allow unimpeded access of all CbMs to their binding sites on the capsid. The anchoring of NUP153 to the nuclear basket would likely constrain the reach of NUP153 and the CbMs into the pore as they are distributed at different positions along the NUP153c. Given the opposing models of capsid nuclear ingress, positioning of CbMs in the NPC could be important for interaction with capsids docked at the surface of NPC or during transit of the intact or remodelled capsid into the cytoplasm [8]. This would be similar to the apparent competitive interactions between HIV cofactors that influence its transport through the NPC; specifically, CPSF6 is required to release the capsid from the NPC [59].

The complementation of NUP153-depleted cells by GFP-NUP153 with a loss-of-function mutation in CbM.1 reduced viral transduction and 2-LTR circles. This is evidence showing that CbM.1 in the context of the NPC is important for HIV nuclear ingress, which is supported by many other published studies. Specifically, these data are consistent with prior RNAi depletion assays showing a block to capsid nuclear ingress upon the loss of NUP153 or treatment with drugs that occlude the NUP153 binding pocket on capsid [18,37, 60]. However, the mutation F1228A in CbM.2, which was predicted to abolish capsid binding by molecular modelling, did not appear to disrupt the HIV replication cycle in the same way as mutations in CbM.1. Indeed, F1228A did not decrease HIV transduction, suggesting that the mutation did not affect reverse transcription or nuclear import of viral, although these were not directly measured. The increase in luciferase signal could suggest that this FG motif is antagonistic to HIV and that its loss increases NUP153 interaction. This is supported by CA hexamer tube binding assay data showing that the deletion of CbM.2 caused an increase in NUP153 binding. However, NUP153 is also important for integration site selection, and F1228A could increase the expression of the luciferase reporter [61]. The surprising increase in 2-LTR circles indicates the effect of F1228A after nuclear ingress. It could indicate a block on HIV integration, which also occurs upon the loss of the host cell protein CPSF6 or treatment with the integrase inhibitor Raltegravir [62,63]. However, the latter does not cause such a large increase in 2-LTR circles relative to the loss of CPSF6.

The effect of F1228A on HIV is similar to the knockdown of CPSF6, where there is an increase in luciferase and a disproportionate increase in 2-LTR circles. However, the observed increase in 2-LTR circles is more dramatic for F1228A [62]. The knockdown of NUP62 also increases 2-LTR circles, but this is accompanied by a loss of viral transduction [20]. Given that loss of CbM.2 phenocopies loss of CPSF6, we speculate that CbM.2 could have an undescribed role in a step prior to capsid release from NPC [62,64, 65]. The knockdown of CPSF6 or using the CPSF6-independent CA mutant A77V arrests the capsid at the NPC, colocalized in the nucleoplasm with NUP153 [64]. Alternatively, NUP153 has also been shown to be important for the interaction with HIV integrase to facilitate nuclear entry, which could be disrupted by the mutation of CbM.2, preventing integration and increasing 2-LTR circles [4]. NUP153 has a role in DNA repair pathways through the nuclear import of 53BP1, which could also be affected by mutations in NUP153 that could influence the formation of 2-LTR circles by the non-homologous end-joining pathway of DNA repair [33,53]. Further investigation of the role of CbM.2 in the HIV replication cycle, specifically in the processes of capsid trafficking and integration target site selection, will be important in determining its functional role beyond the direct binding of the HIV capsid reported in this manuscript.

## Supplementary material

10.1099/jgv.0.002104 Uncited Supplementary Material 1.

## Abbreviations

CbM.1: capsid-binding motif 1
CbM.2: capsid-binding motif 2
CbM.3: capsid-binding motif 3
CbM.4: capsid-binding motif 4
DMEM: Dulbecco’s Modified Eagle Medium
DPBS: Dulbecco’s PBS
FG: phenylalanine-glycine
HIV: human immunodeficiency virus
MD: molecular dynamics
NPC: nuclear pore complex
NUP153: nucleoporin 153
NUP153c: C-terminal domain of NUP153
RLU: relative light unit
RMSF: root mean square fluctuation
SPR: surface plasmon resonance
TBST: TBS containing 0.1% Tween 20
