# Supplementary material for: The identification of a novel interaction site for the human immunodeficiency virus capsid on nucleoporin 153
Source: J Gen Virol. 2025 May 14;106(5):002104. doi: 10.1099/jgv.0.002104 (PMC12078792; doi:10.1099/jgv.0.002104)
Supplement: Uncited Supplementary Material 1. [file jgv-106-02104-s001.pdf]

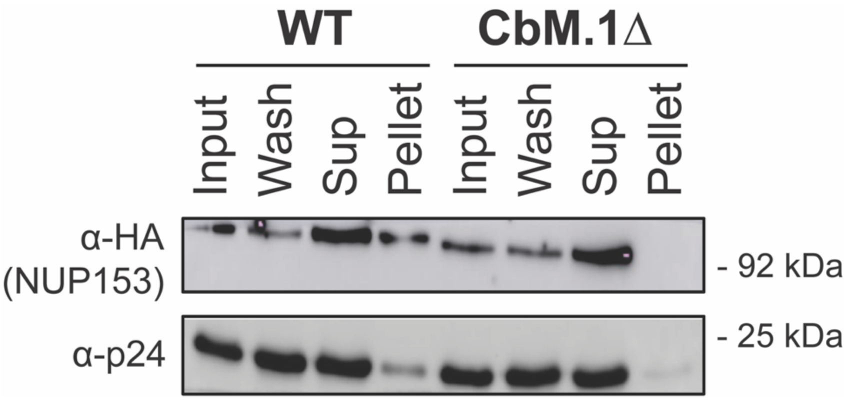

Figure S1. Co-sedimentation of capsid tubes and NUP153C wild-type or CbM.1∆ mutant with ß-mercaptoethanol. The co-sedimentation of wild-type NUP153c and CbM.1∆ with HIV CA tubes. Western blotting detected HIV p24 and HA-tagged NUP153c in the whole cell extract (Input), supernatant (Sup), Wash, and Pellet fractions. To obtain NUP153c, HEK293T cells were transfected with 1 ug plasmid per well (200,000 cells) and the protein content of lysates for western blotting was normalized by Bradford assay to ~4 μg/well. The blot is a representation of three independent biological replicate blots.

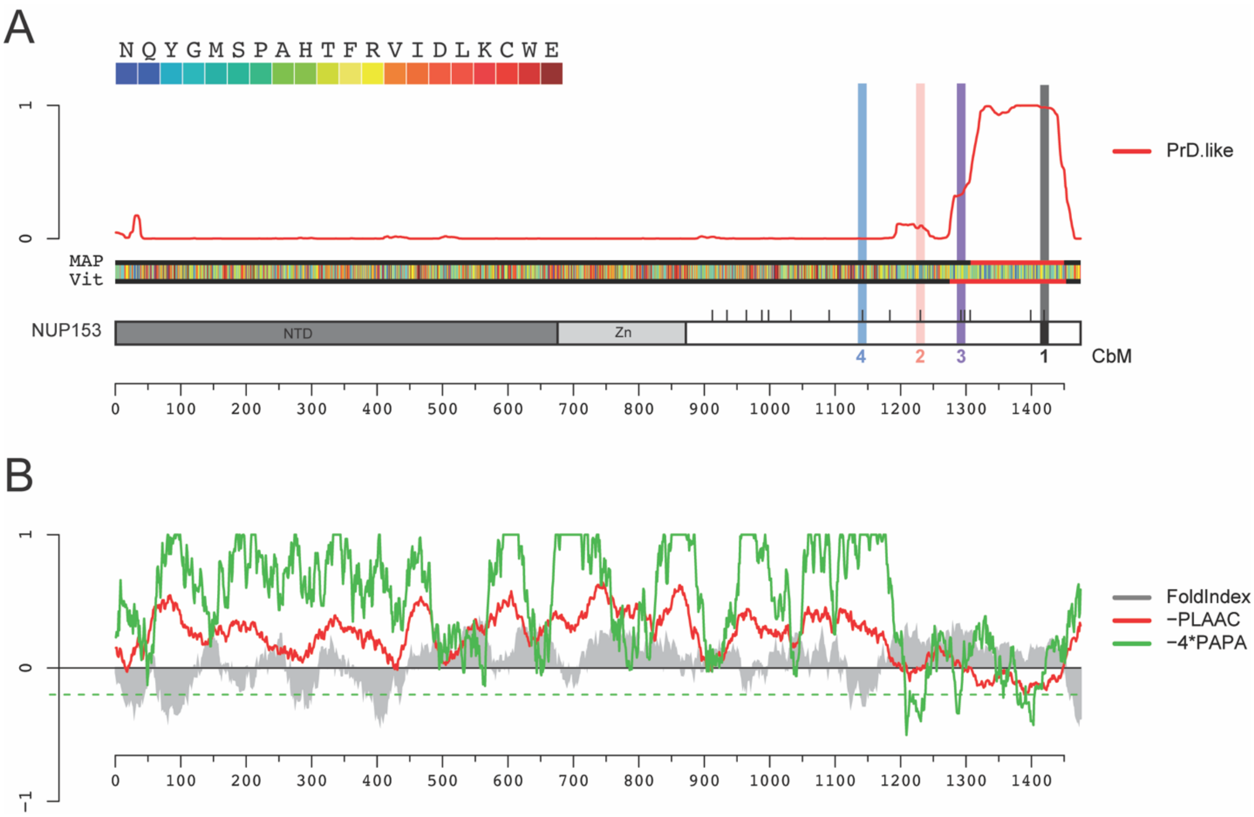

Figure S2. PLAAC analysis of NUP153. (A) Predicted probability of prion-like state formation across NUP153. MAP and Vit denote the Maximum a Posteriori and the Viterbi parses of NUP153 into the prion-like state (indicated by the red shading). Colored lines between MAP and Vit plots indicate the amino acid composition as colored in the key. Analysis is placed in the context of NUP153 and the four CbM. Tick marks on the domain diagram of NUP153 represent FxFG motifs. Zn - zinc finger domain. NTD - N-terminal domain. (B) A representation of the predicted disorder of NUP153 in gray, as well as the PLAAC and PAPA scores.

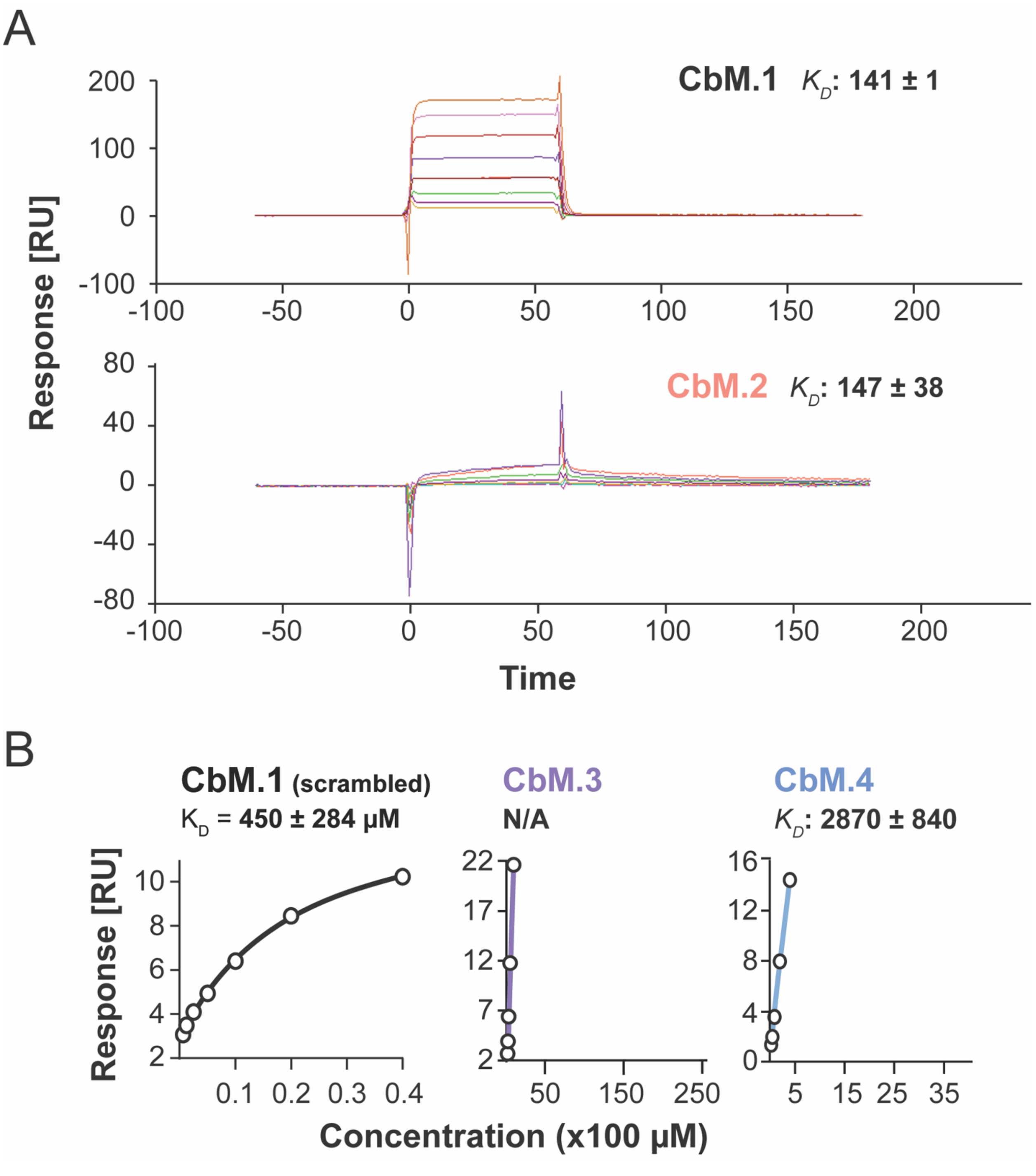

Figure S3. SPR analyses of CbM peptides binding with recombinant CA hexamers. (A) Raw SPR sensorgram data for the binding of CbM.1 and CbM.2 that was used to generate figure 3A. (B) SPR analyses of scrambled CbM.1, CbM.3, and CbM.2 peptides that bound weakly to CA hexamers or could not be accurately calculated (N/A). Each measurement series was repeated three times. The model calculated the equilibrium dissociation constant K_D for the 1:1 binding. Error: standard deviation (n=3, biological replicates).

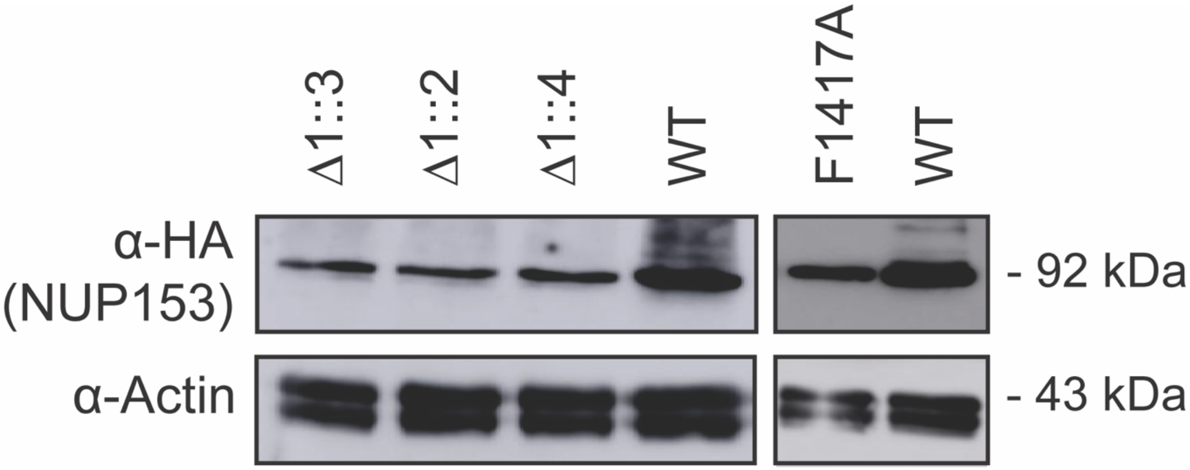

Figure S4. Expression of TRIM-NUP153C mutants stably expressed in CRFK cells assayed by western blotting. Western blotting detected HA-tagged NUP153c and actin in the whole cell extracts. CRFK cells stably expressing wild-type NUP153c and mutants were selected for over two weeks with puromycin (3 μg/mL) before being used in assays. Typically, 200,000 cells were assayed with ~5 μg total amount of protein lysates each cell line.

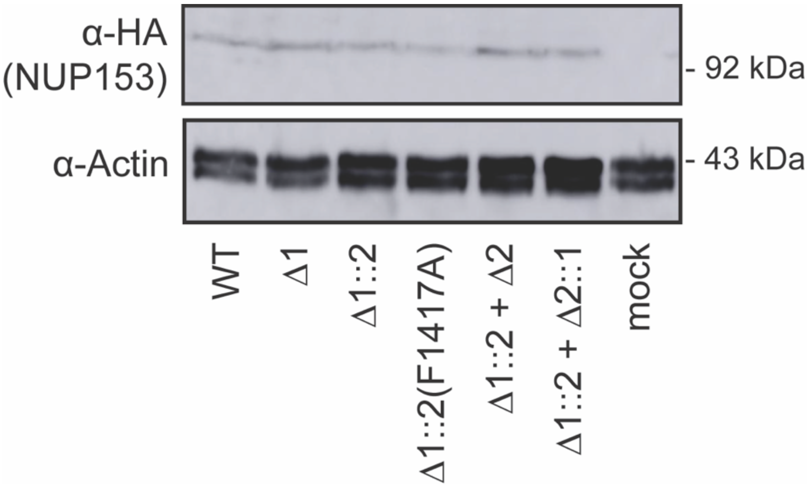

Figure S5 Expression of TRIM-NUP153c with mutations in CbM.1 and CbM.2: Western blot of TRIM-NUP153c mutants transiently expressed in HEK293T cells 24 hours post-transfection. NUP153c and mutants were obtained by transfecting 200,000 HEK293T cells each well with 1 μg plasmid and the protein content of lysates for western blotting was normalized by Bradford assay to ~4 μg/well. The blot is a representation of three independent biological replicate blots

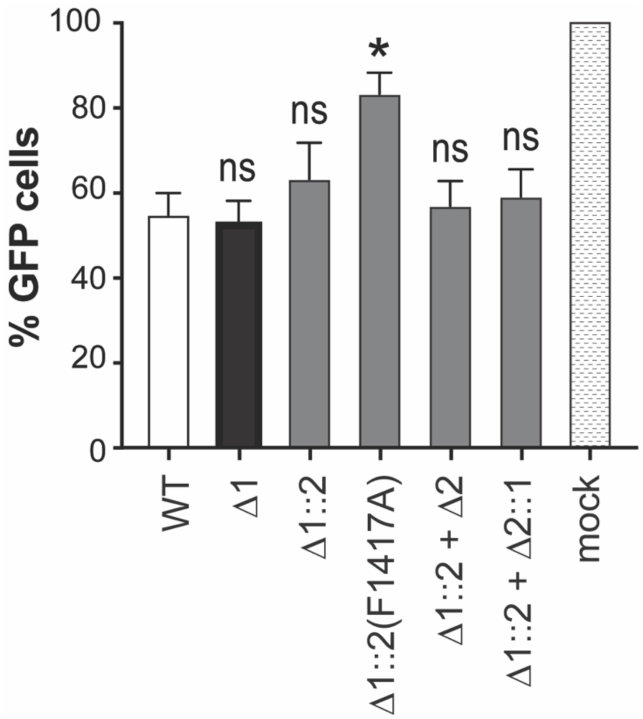

Figure S6. Restriction of EIAV by TRIM-NUP153c with mutations in CbM.1 and CbM.2. (A) A cell-based restriction assay with transient expression of TRIM-NUP153c mutants transduced by EIAV-GFP. Virus transduction was measured relative to a mock-infected control. Error bar: standard error. Statistical analysis: one-way ANOVA followed by a Tukey's test (n=3). Asterisk: significantly different from WT. ns: not significant.

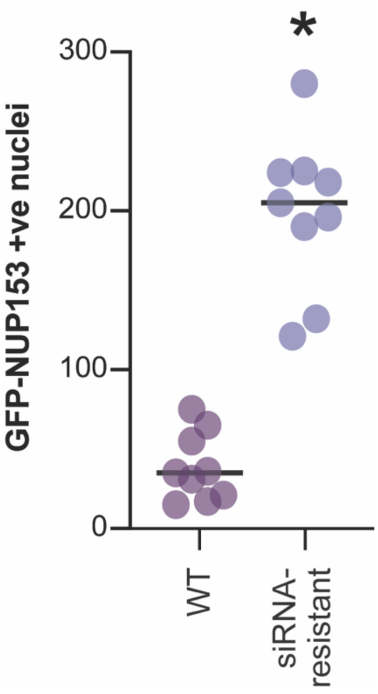

Figure S7. Expression of GFP-NUP153 designed to resist siRNA knockdown. HeLa cells with the endogenous NUP153 were knocked down and complemented with plasmids encoding GFP-NUP153. The complementing gene sequence of NUP153 was either the wild-type nucleic acid sequence or a mutant sequence with synonymous mutations designed to prevent recognition by a siRNA targeted to NUP153. After transfection with both siRNA and the complementing plasmids the number of GFP-positive nuclei were counted as a measure of the successful expression of GFP-NUP153. Asterisk: significantly different from WT (student's t-test).

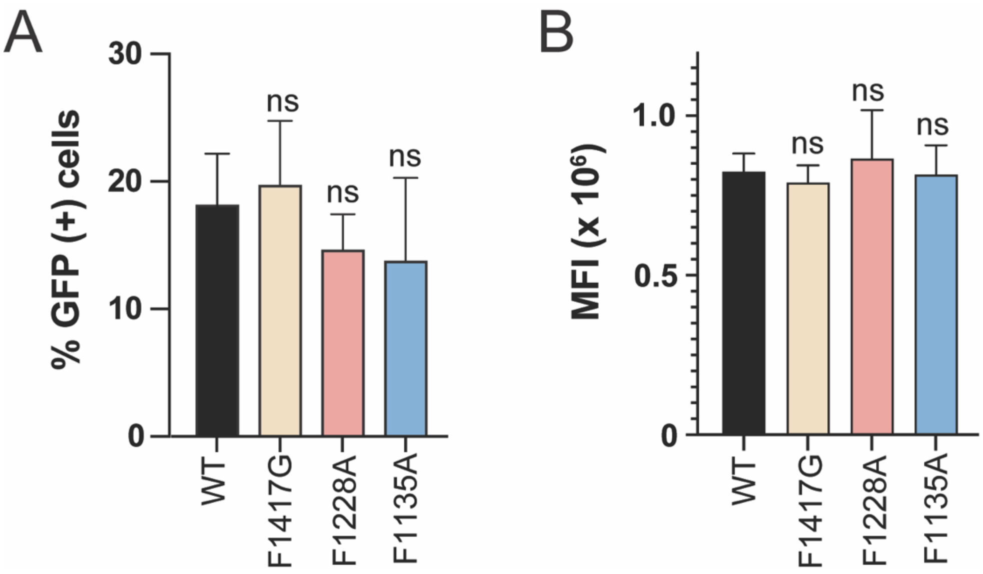

Figure S8 Expression of NUP153-GFP constructs in HeLa cells measured by flow cytometry. (A) Overall GFP positive cells when HeLa cells (endogenous NUP153 knockdown) were transfected with GFP-NUP153 WT, F1417G, F1228A, or F1135A mutants. (B) Mean fluorescent intensity (MFI) of the GFP positive HeLa cells. ns: not significant from WT (Tukey's test).

Oligo	Sequence (5' - 3')	Notes
SL043	TGGTTTCGGCgctGGAGCCACAAC	F1291A FORWARD
SL044	GAGGTGGTAGTATTATTACTGC	F1291A reverse
SL047	TACCTTTGTGgctGGACAGTCCAGC	F1228A forward
SL048	GCCACAGGTGGATTGGAG	F1228A reverse
SL055	CAGTCCAGCAATCCTGTG	Nup153 motif-3 KO forward
SL056	TGGATTGGAGGAAGAGGTG	Nup153 motif-3 KO reverse
SL057	AATTCAGAGCAAACCAAAG	Nup153 motif-4 KO forward
SL058	TTGACACTTTGGCTCTTC	Nup153 motif-4 KO reverse
SL059	GCCACAACCACATCTAGC	Nup153 motif-2 KO forward
SL060	GGTAGTATTATTACTGCTGGC	Nup153 motif-2 KO reverse
SL063	AGTGTTCACAgctGGTGCAAATTCTAG	Nup153 F1417A forward
SL064	CCTGATGGACTGTTGTTTG	Nup153 F1417A reverse
SL139	GCAAATTCTAGCACACCTG	Nup153 motif-1 KO forward

SL140	ACTGTTGTTTGTGAAGTTG	Nup153 motif-1 KO reverse
SL141	ttcggctttggaGCAAATTCTAGCACACCTG	Motif-2 replacing motif-1 Forward
SL142	accagaggtggtACTGTTGTTTGTGAAGTTG	Motif-2 replacing motif-1 reverse
SL143	tttgtgtttggaGCAAATTCTAGCACACCTG	Motif-3 replacing motif-1 Forward
SL144	ggtagccacaggACTGTTGTTTGTGAAGTTG	Motif-3 replacing motif-1 reverse
SL145	tcctttgggGCAAATTCTAGCACACCTG	Motif-4 replacing motif-1 Forward
SL146	aaacactggACTGTTGTTTGTGAAGTTG	Motif-4 replacing motif-1 reverse
SL189	CAGTCCAGCAATCCTGTG	Motif-1 replacing motif-3 (3/1 swapping) forward
SL190	cactcctgatggTGGATTGGAGGAAGAGGTG	Motif-1 replacing motif-3 (3/1 swapping) reverse
SL221	aaatcgacttCTGTTGCTGCTCAGCCCA	pEGFP(C3)-Nup153 Moudry siNup153 resistant forward
SL222	tgggggaggcAAACCAGGGCTTTTCAGAATATCTAG	pEGFP(C3)-Nup153 Moudry siNup153 resistant reverse

Table S1. Oligonucleotides used in this study.

Plasmid ID	Specified	Origin
pUI034	pCR8-TRIM-Nup153C (Human)	This paper
pUI020	pCDNA3-TRIM-Nup153C (Human)	This paper
pSL072	pCR8-TRIMNUP153c Motif-4/-1 DKO	This paper
pSL091	pCR8-TRIMNUP153c Motif-1 KO (minimal)	This paper
pSL092	pCR8-TRIMNUP153c Motif-2 replacing motif-1 (double motif-2)	This paper
pSL093	pCR8-TRIMNUP153c Motif-3 replacing motif-1 (double motif-3)	This paper
pSL094	pCR8-TRIMNUP153c Motif-4 replacing motif-1 (double motif-4)	This paper
pSL095	pLPCX-TRIMNUP153c Motif-2 replacing motif-1 (double motif-2)	This paper
pSL096	pLPCX-TRIMNUP153c Motif-3 replacing motif-1 (double motif-3)	This paper
pSL097	pLPCX-TRIMNUP153c Motif-4 replacing motif-1 (double motif-4)	This paper
pSL099	pCR8-TRIMNUP153c delta Motif-3 Motif-3	This paper
pSL102	pCR8-TRIMNUP153c Motif-1 Motif-3	This paper
pSL103	pCDNA3-TRIMNUP153c Motif-3 replacing motif-1 (double motif-3)	This paper
pSL104	pCDNA3-TRIMNUP153c Motif-4 replacing motif-1 (double motif-4)	This paper
pSL105	pCDNA3-TRIMNUP153c Motif-5 replacing motif-1 (double motif-5)	This paper
pSL106	pCR8-TRIMNUP153c Motif-4 replacing motif-1 (double motif-4) FA	This paper

pSL107	pCDNA3-TRIMNUP153c delta Motif-4 Motif-4	This paper
pSL111	pCDNA3-TRIMNUP153c Motif-1 Motif-4	This paper
pSL112	pCR8-Nup153C F1417A	This paper
pSL114	pCDNA3-TRIMNUP153c Motif-4 replacing motif-1 (double motif-4) FA	This paper
pSL116	pET11A-CA-089-N57A	This paper
pSL117	pET11A-CA-121-N57A	This paper
pSL122	pEGFP(C3)-Nup153	Addgene
pSL123	pCDNA3-TRIMNUP153c Motif-1 KO (minimal)	This paper
pSL126	pCR8-TRIMNUP153 Motif-1/-3 DKO	This paper
pSL128	pET11A-CA-089	Dr. Pornillos
pSL129	pET11A-CA-121	Dr. Pornillos
pSL131	pSPax2	Dr. Fortunato
pSL132	pLJM1-eGFP	Dr. Fortunato
pSL133	pCR8-TRIMNUP153c #2#1 DKO	This paper
pSL135	pCR8-TRIMNUP153c #3#1 DKO	This paper
pSL136	pEGFP(C3)-Nup153 siRNA resistant	This paper
pSL141	pCR8-TRIMNUP153c #4#1 DKO	This paper
pSL142	pCDNA3-TRIMNUP153c #2#1 DKO	This paper
pSL143	pCDNA3-TRIMNUP153c #3#1 DKO	This paper
pSL145	pEGFP(C3)-Nup153 siRNA resistant F1417G	This paper
pSL146	pEGFP(C3)-Nup153 siRNA resistant F1228A	This paper
pSL147	pEGFP(C3)-Nup153 siRNA resistant F1417G/F1228A	This paper
pSL150	pEGFP(C3)-Nup153 siRNA resistant F1291A	This paper
pSL151	pEGFP(C3)-Nup153 siRNA resistant F1135A	This paper

Table S2. Plasmids used in this study.
